# Intravenous Leiomyomatosis Complicated by Arteriovenous Fistula: Case Series and Literature Review

**DOI:** 10.3389/fcvm.2022.878386

**Published:** 2022-06-13

**Authors:** Haoxuan Kan, Yang Cao, Yuexin Chen, Yuehong Zheng

**Affiliations:** ^1^Peking Union Medical College Hospital, Chinese Academy of Medical Sciences and Peking Union Medical College, Beijing, China; ^2^Peking Union Medical College, Chinese Academy of Medical Sciences (CAMS), Beijing, China

**Keywords:** arteriovenous fistula (AVF), vascular surgery, coil embolization, pelvic mass, intravascular leiomyomatosis (IVL)

## Abstract

**Background:**

Uterine intravenous leiomyomatosis (IVL), a rare type of uterine leiomyoma, is defined by the intravascular proliferation of a histologically benign smooth muscle cell tumor. Pelvic arteriovenous fistula (AVF) is a rare vascular malformation that is most commonly congenital, post-traumatic, or iatrogenic. The link between leiomyomatosis and AVF has received little attention in the medical literature.

**Results:**

We provide a case series of seven patients, four of whom were from our center, who had IVL complicated by a pelvic AVF. The symptoms of right heart failure were noted as swelling in the abdomen and two legs as well as a significant amount of ascites. Coil embolization of AVFs may be beneficial in minimizing bleeding during IVL surgery. A review of all accessible literature published on IVLs from 2000 to 2020 was conducted, and data were retrieved from 78 papers totaling 262 cases. Complications and recurrence were associated with pelvic mass excision and intravascular remnant tumor, respectively.

**Conclusion:**

Intravenous leiomyomatosis combined with AVF aggravates congestion symptoms of surrounding organs. It is worth noting the uncommon combination of AVF and IVL, stressing the importance of a thorough assessment and surgical approach in IVL treatment.

## Introduction

Uterine intravenous leiomyomatosis (IVL), a rare neoplasm defined by the intravascular proliferation of a histologically benign smooth muscle cell tumor, is an uncommon growth pattern of uterine leiomyoma (1). The clinical course varies depending on the severity of the condition. Vascular smooth muscle tumors can spread into the major veins and even the right atrium of the heart, obstructing blood flow and causing death (2–5).

Appropriate imaging examinations are required since IVLs could be misdiagnosed as intravascular thrombus, myxoma, or pancreatic tumors (6–8). Enhanced CT imaging can show the location, size, and full-scale extension pathway of IVL lesions, and it can be utilized in preoperative assessment (9). MRI could help with an accurate diagnosis, which is critical for deciding on a surgical plan and achieving a positive outcome (6). Echocardiography can be used to assess extension into the right atrium (10).

Pelvic arteriovenous fistula (AVF) is a rare vascular malformation that is most commonly congenital, post-traumatic, or iatrogenic (11, 12). Massive AV shunting causes high output cardiac failure. This malformation is hard to eradicate completely because of a high recurrence rate. Because of the significant hemorrhage, surgical resection is often challenging. Although transcatheter embolization has recently become the treatment of choice for pelvic AVF, full embolization to stop the shunt flow is equally challenging (11–13). The link between leiomyomatosis and AVF has only been mentioned rarely in the medical literature. Three definite cases have been recorded since the first description in 1993 (14–16). We provide four further examples of fistula associated with leiomyomatosis, review the literature on the topic, and speculate on possible pathophysiological causes for the cooccurrence.

## Methods

For the literature review, PubMed, Embase, CNKI, and WanFang were utilized to conduct systematic searches of peer-reviewed literature published between 2000 and 2020. IBM SPSS was used to conduct the statistical analysis (IBM SPSS 26.0, SPSS Inc). For qualitative variables, Fisher's exact test and Pearson's chi-square test were utilized. All tests of statistical significance were two-sided, with *p* < 0.05.

## Results

### Case Series

A total of seven patients, four of whom were admitted to Peking Union Medical College Hospital from 2018 to 2020, were discovered to have IVL combined with AVF. Cases 1, 4, 5, 6, and 7 were found to have developed IVL and AVF at the same time, while in cases 2 and 3, AVF was revealed a few years after IVL surgeries.

**Figure 1:**
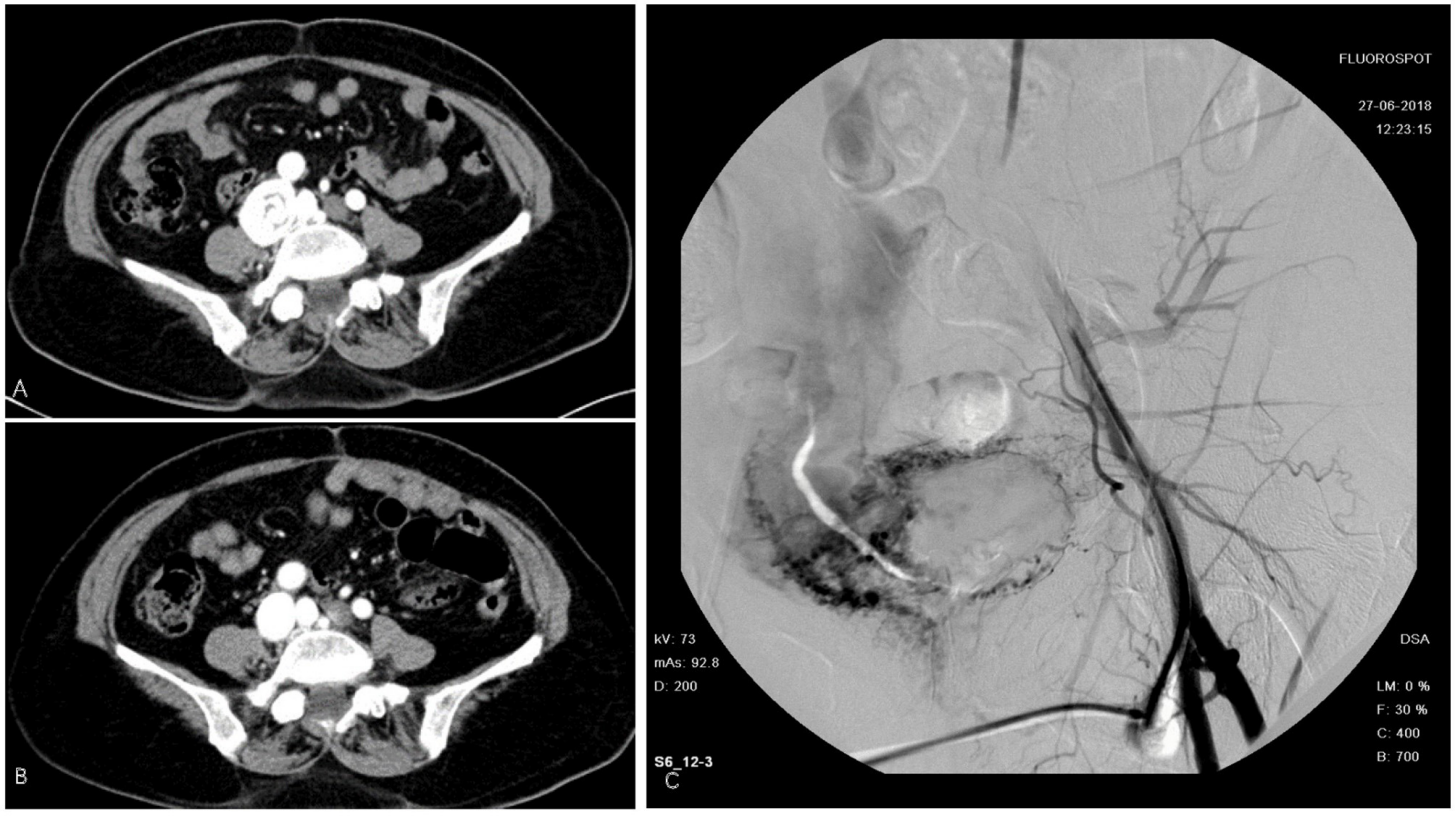
Intravenous leiomyomatosis (IVL) in inferior vena cava (IVC). **(A)** Cord like tumor in IVC. **(B)** Tumor removed surgically. **(C)** Digital subtraction angiography (DSA) demonstrated a contrast agent entering the right iliac vein and IVC through vascular malformation.

The first patient was a 58-year-old woman who was admitted with a 2-month history of right lower limb swelling. IVL was found incidentally 7 months before. Her past medical history included leiomyoma, which was treated by total abdominal hysterectomy and bilateral salpingo-oophorectomy (TAH-BSO). Abdomen-pelvic enhanced CT showed a pelvic mass with abundant blood supply invading the right internal iliac vein, right common iliac vein, and soft tissue in the pelvis. Digital subtraction angiography (DSA) demonstrated multiple iliac AVF (Figure 1). After gynecological consultation, the patient underwent laparotomy and right common iliac venous tumor excision. A mass 2 cm in diameter and 10 cm in length was removed. However, the residual tumor within the right internal iliac vein was left untreated because of a tendency to bleed. On outpatient follow-up in 6 months, IVL recurrence was revealed by ultrasound.

**Figure 2:**
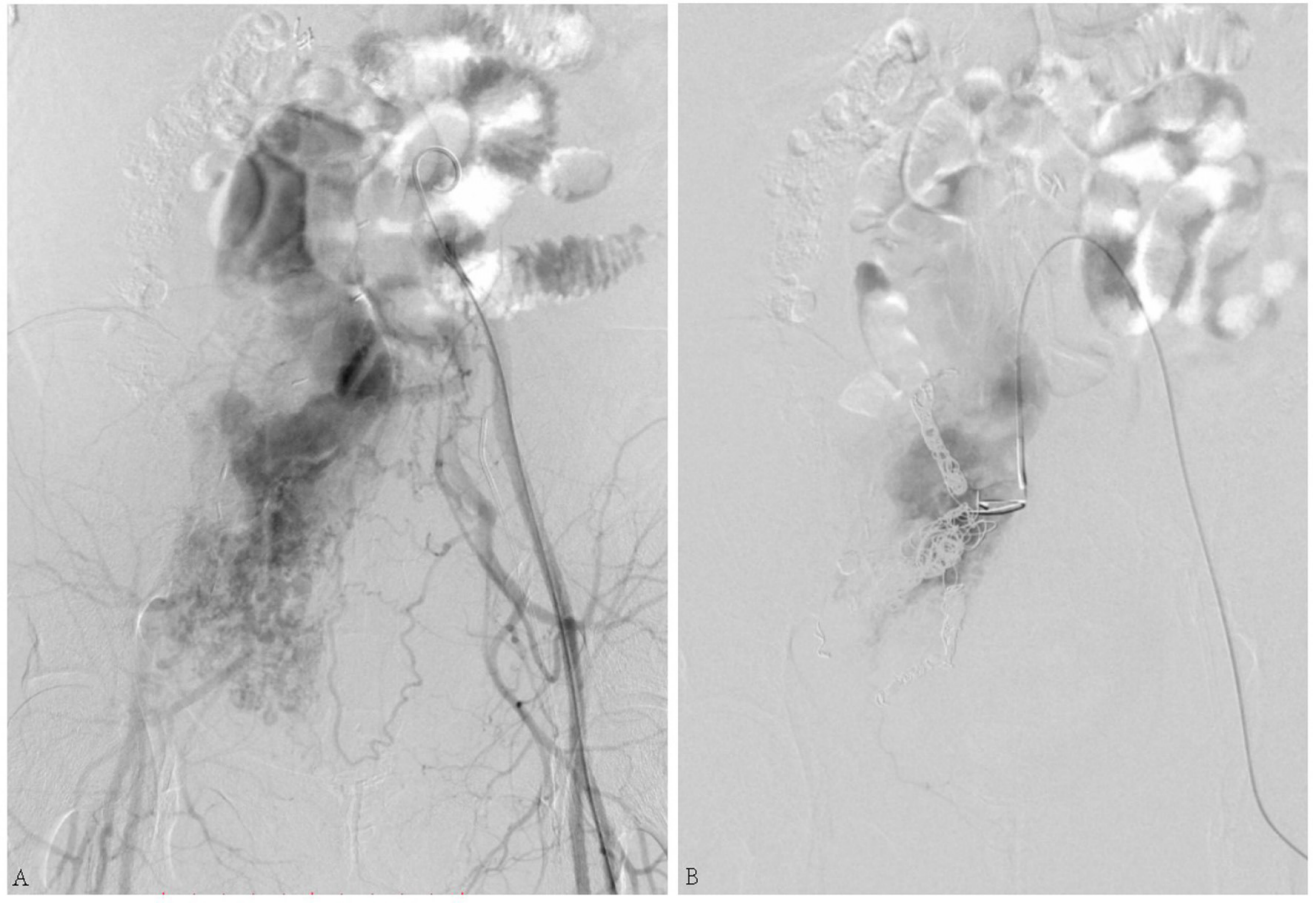
**(A)** Arteriovenous fistula (AVF) connecting the right internal iliac artery and internal iliac vein. **(B)** Shunt flow disappeared after coil embolization.

The second patient was a 49-year-old female who complained of swelling of the abdomen and two legs for 12 months. Iliac AVF was found 2 months before. Her past medical history included leiomyoma, which was treated by hysterectomy and left oophorectomy 17 years ago. She underwent vascular surgery for IVL 3 years before. Echocardiography (Figure 2) revealed enlargement of both atria and right ventricle. The arterial phase during angiography shows the enormous dilatation of both uterine and ovarian arteries to accommodate the high-volume shunting through the pelvic AVF. Coil embolization for pelvic AVF was performed successfully. On outpatient follow-up in 4 months, her condition was favorable and her heart function improved.

**Figure 3:**
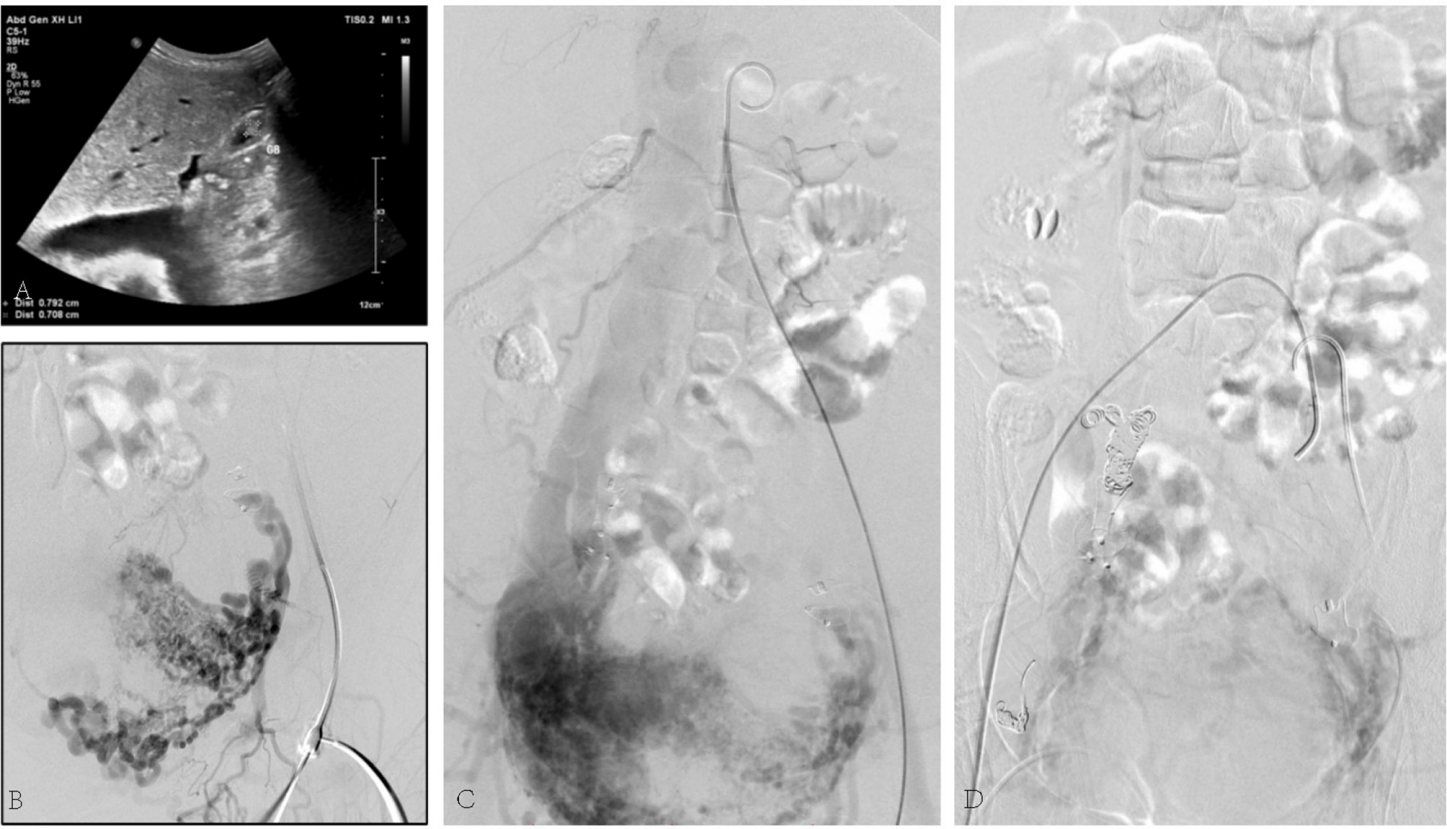
**(A)** Ultrasound revealed congested liver. **(B)** Arteriovenous malformation. **(C)** Shunt flow entering the vein. **(D)** Abnormal flow stopped by the placement of embolization coils.

The third case was a 45-year-old female suffering from a large number of ascites. Her skin and mucosa were yellow. A vascular murmur could be heard in both femoral regions. The medical history was complicated by myomectomy and coil embolization for bilateral iliac aneurysms 8 years before. An ultrasound revealed enlarged liver. Pelvic AVF with high-volume shunting is obvious in angiography (Figure 3). She was treated with coil embolization. However, the iliac AVF still existed after 5 months.

**Figure 4:**
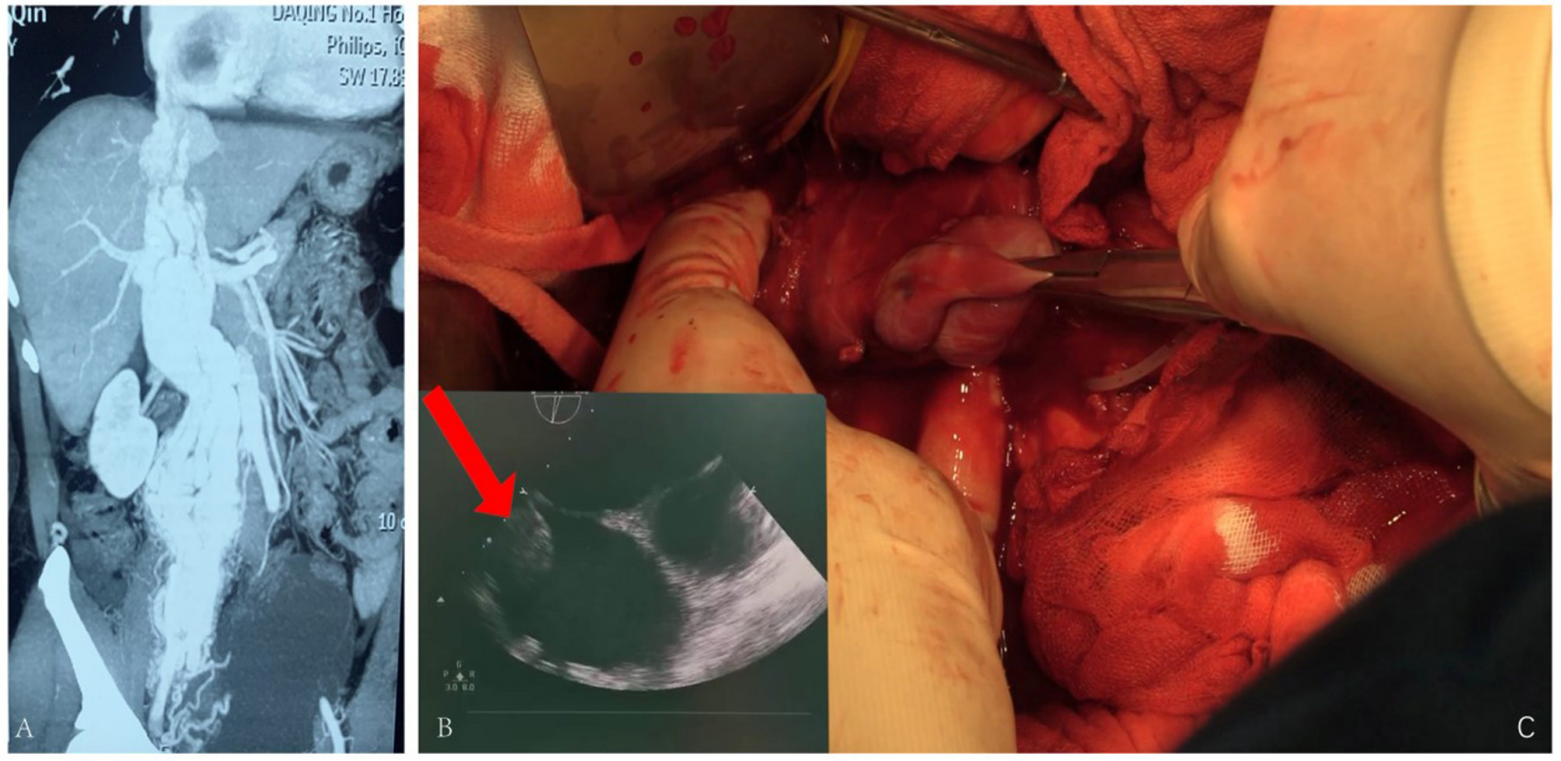
**(A)** Tumor enhanced in the arterial phase. **(B)** Ultrasound revealing tumor being extracted during surgery. **(C)** Tumor being pulled out from the IVC.

The fourth case, 50 years old, had edema of both lower limbs, coughing, and blood-stained sputum for 3 months. She had a hysterectomy with right oophorectomy for over 7 years. Doppler ultrasound of iliac arteries and veins, echocardiography, PET-CT, and enhanced pelvic MRI were carried out (Figure 4), showing a giant tumor with enhancement in her inferior vena cava (IVC) and right atrium. The tumor was successfully extracted through IVC incision, yet the pelvic mass was not removed due to massive blood supply. Bilateral internal iliac veins were ligated to reduce the arteriovenous shunt flow. The patient recovered well.

After a thorough literature search, three case reports with a combination of AVF and IVL were found (Table 1). Lee et al. reported the first case in which high-output cardiac failure was caused by the development of arteriovenous shunting within the intravenous component of the tumor. Treatment by TAH-BSO and tumor mass excision was successful. This report pointed out that AVF could be formed within the tumor of leiomyomatosis. This was also shown in a few case reports, where arteries within the tumor of IVL revealed by computed tomography angiography (CTA) are parallel with the vena cava (5, 14).

**Table 1:** Review of intravenous leiomyomatosis (IVL) combined with arteriovenous fistula (AVF).

**Publication**	**Past Medical History**	**IVL Location**	**AVF Location**	**Treatment**		**Outcome**
Lee et al. (14)	/	Retroperitoneal and Ovarian veins	Retroperitoneal and Overian veins	IVL excision TAH-BSO	/	/
Nishizawa et al. (15)	Hysterectomy	Left Ovarian Vein	Bilateral RA Lumbar arteries Right IIA	IVL partial excision Hormonal therapy	AVF untreated	No recurrence
Mizuno et al. (16)	Caesarian operation Hysterectomy	Left Ovarian Vein	Right IIA	IVL excision TAH-LSO	AVF surgically removed	No recurrence

Nishizawa et al. reported the second case. The patient had received a hysterectomy, whose AVF was extensive and was involved with the intravenous tumor. Therefore, the surgery was in danger of massive hemorrhage. In the end, the tumor was only partially resected, and AVF was left untreated (15).

Mizuno et al. reported the third case of lVL associated with a pelvic AVF. The patient had received a caesarian operation and a hysterectomy. However, the patient had a separate pelvic AVF, which was not associated with IVL. Besides, the IVL was complicated by intracardiac extension. During the surgery, bleeding was hard to control due to the high venous return from the pelvic AVF at the time of removing the intravenous tumor (16). In these cases, AVF was found at the same time when IVL was diagnosed, yet few treatments were done for the AVF.

### Literature Review

**Figure 5:**
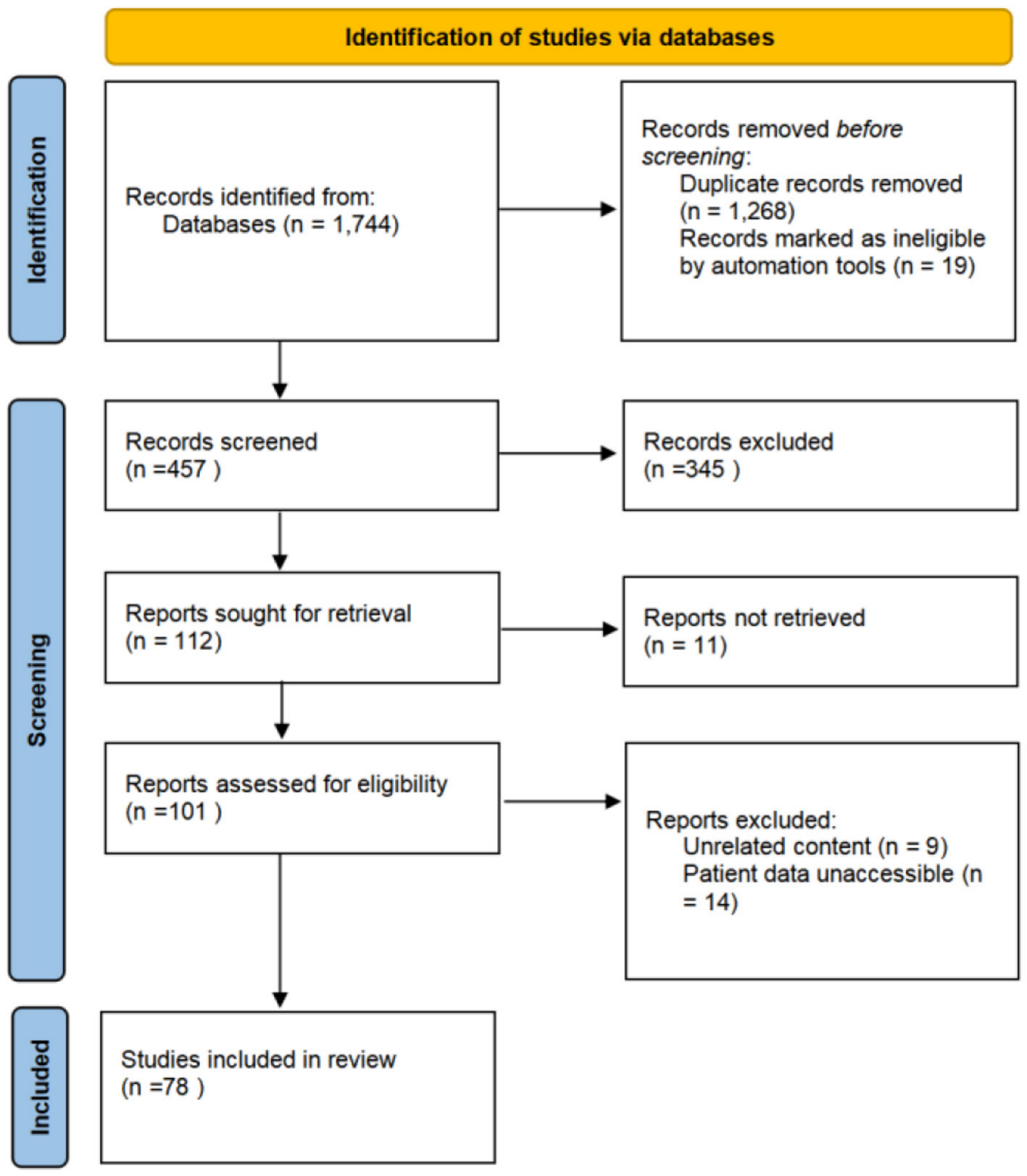
The Preferred Reporting Items for Systematic Reviews and Meta-Analyses (PRISMA) diagram of study screening for the literature review. A total of 1,744 studies were identified, from which 78 were included in the review.

A total of 457 articles on IVLs were retrieved from the initial search from 2000 to 2020 (Figure 5). A total of 78 documents were chosen for complete analysis after abstract screening and full-text reviews, reporting a total of 262 cases.

Asian patients accounted for the majority of all reported IVL cases in literature. Cardiac involvement is one of the most common characteristics in patients with IVL, which could be found in 80.5% of all cases. Surgery is the primary strategy to remove IVL, both one-stage and staging surgeries could be considered according to patient condition and tumor shape. It should be highlighted that the rate of misdiagnosis on IVLs was high, with 17 cases reported, accounting for 6.5% of total cases (Table 2).

**Table 2:** Summary of the clinical information of 262 patients with IVL.

**Patients**	262	**Involving vessel**	
**Age**	46.01 ± 7.13	Ovarian vein	69 (26.3%)
**Race**		Uterine vein	20 (7.6%)
Asian	189 (72.1%)	Internal iliac vein	80 (30.5%)
Caucasion	70 (26.7%)	Common iliac vein	13 (5.0%)
African	3 (1.1%)	Inferior vena cava	2 (0.8%)
**Reproductive history**		Nephrotic vein	5 (1.9%)
Yes	25 (9.5%)	NR	73 (27.9%)
None	9 (3.4%)	**Cardiac involvement**	
NR	228 (87.0%)	Yes	211 (80.5%)
**History of fibroids**		No	47 (17.8%)
Yes	161 (61.5%)	NR	4 (1.7%)
None	13 (5.0%)	**Tumor enhanced**	
NR	88 (33.6%)	Yes	8 (3.1%)
**Myomectomy history**		No	24 (9.2%)
Yes	113 (43.1%)	NR	230 (87.8%)
None	30 (11.5%)	**Surgery Staging**	
History of IVL surgery	15 (5.7%)	One-stage surgery	185 (70.6%)
NR	104 (39.7%)	Staging surgery	53 (20.2%)
**Symptoms**		Non-operative	3 (1.1%)
Dyspnea	87 (33.2%)	NR	21 (8.0%)
Palpitation	58 (22.1%)	**Cardiotomy**	
Chest pain	39 (14.9%)	Yes	118 (45.0%)
Syncope	40 (15.3%)	No	124 (47.3%)
Fatique	10 (3.8%)	NR	20 (7.6%)
Abdominal discomfort	41 (15.6%)	**Complete excision of intravascular mass**	
Lower limbs swelling	48 (18.3%)	Yes	138 (52.7%)
Pelvic mass	53 (20.2%)	No	30 (12.2%)
Menorrhagia	26 (9.9%)	NR	95 (35.1%)
None	34 (13.0%)	**Excision of pelvic mass**	
**Imaging**		Yes	191 (72.9%)
Enhanced CT	53 (20.2%)	No	11 (4.2%)
MRI	40 (15.3%)	NR	60 (22.9%)
CT	62 (23.7%)	**Complication**	
ECHO	67 (25.6%)	Yes	50 (19.1%)
Pelvic ultrasound	20 (7.6%)	No	54 (20.6%)
Laparoscopy	4 (1.5%)	NR	161 (66.8)
Others	10 (3.8%)	**Recurrence**	
NR	83 (31.7%)	Yes	18 (7.5)
		No	93 (38.6)
**Misdiagnose**	17 (6.5%)	NR	130 (53.9)

Intravenous leiomyomatosis recurrences are prevalent. However, complete excision of intravascular mass is associated with a reduced recurrence rate (*p* < 0.05). Nevertheless, the incidence of complications was increased with complete excision, though not significantly compared with non-complete excision (Table 3). In contrast, intrapelvic mass resection might increase the incidence of complications (*p* < 0.01) but did not significantly reduce IVL recurrence. It is crucial to highlight that these comparisons have relatively little statistical efficacy, and do not reveal which types of patients would have severe complications and die.

**Table 3:** Factors associated with complications and recurrence of IVL.

		**Complications**	***p*** **value**	**Recurrence**	***p*** **value**
Reproductive history	Yes	7/11	1.000	4/21	1.000
	No	3/5		1/7	
History of fibroids	Yes	17/28	0.295	8/40	1.000
	No	1/4		0/4	
History of myomectomy or IVL	Yes	9/19	1.000	7/36	0.659
	No	4/10		1/11	
Complete excision of intravascular mass	Yes	18/28	0.236	7/53	0.024^*^
	No	3/8		6/10	
Excision of pelvic mass	Yes	30/42	0.007	11/49	0.673
	No	1/7		3/9	
Tumor enhanced	Yes	2/4	1.000	1/5	1.000
	No	6/9		1/9	
Cardiac involvement	Yes	11/28	0.062	9/49	0.500
	No	7/9		5/18	

*Fisher's exact test,*

* *Calculated using Pearson χ2*.

## Discussion

The cause of AVFs formation in patients with IVL is unknown, and the association between AVF and IVL cannot be confirmed. Spontaneously developed pelvic vascular malformations are rare, and the most common causes include trauma, iatrogenic injury, aneurysm, and malignant tumor-related neovascularization (13, 17, 18). Vascular malformation induced by benign tumors, such as IVL, is seldom reported.

As demonstrated in cases 1 and 4, secondary vascular abnormalities developed around the tumor at the site of IVL invading the vein for unexplained reasons. The contrast agent in the vein appeared in advance under the angiography, indicating that the process of IVL invading the vein may promote local vascular malformation. Sometimes, this vascular malformation can even become the primary diagnosis in patients, masking IVL and leading to missed diagnosis and ignorant of the leiomyomatosis, resulting in tumor development (19).

There are several possible reasons why AVF would form in these patients with IVL. Six patients, including four cases from our center, who had leiomyomatosis complicated by pelvic AVF had received uterine surgery 7–15 years before. Thus, Iatrogenic AVF is to be suspected (15, 16). Furthermore, leiomyomatosis exhibits behaviors resembling malignant tumors; it invades blood vessels and induces angiogenesis. If the tumor invades and destroys both veins and arteries, fistulas could be formed during angiogenesis. AVF may originate from the nutritional artery of the tumor itself, and form AVFs as the tumor invades and grows into the vein. Such a process might be induced by iatrogenic injury during uterine surgery (20). The tumor inside the vein blocks the blood return, increasing local pressure, resulting in sphincter relaxation, and remodeling of the AVF. Some fistulas regressed after pressure relief (21, 22). The observation of pulmonary vascular malformation induced by benign metastasizing leiomyoma (BML) of the lungs increases the possibility of the hypotheses above.

Although pathological research provided evidence for AVF formation in the myometrium, such theories lack the support of long-term imaging surveillance (23, 24). Considering the risk of iatrogenic AVF, gynecologists are advised to avoid injury to arteries and veins during pelvic surgery. Among the reported cases and those reported by us, there were three patients [Case 2~3, and Mizuno et al. (16)] whose AVF and IVL occurred at different sites, which may have other reasons.

Magnetic resonance angiography (MRA) and DSA should be considered for the evaluation in IVL patients with heavy right cardiac load to exclude potential AVF. The advantage of MRA lies in its better ability to differentiate between soft tissue vascular malformations and soft tissue masses (25). Compared with CT, especially in patients with IVL, MRA can reduce the probability of misdiagnosing IVL as venous dissection or other hyper-vascular soft tissue tumors (19). The advantage of angiography is that it can clearly diagnose vascular malformations, which can be treated with coil embolization during the examination (11, 19). If a vascular malformation is found, it should be embolized from the arterial segment before the IVL surgery to reduce the blood flow in the vascular malformation and prevent bleeding during the IVL surgery, which contributes to a safe operation. If the vascular malformation is discovered during the operation, it is often difficult to remove due to tight adhesion and severe bleeding, as in Case 1. At this time, ligation of the artery supplying the AVF should be considered, as in Case 4.

Vascular malformation aggravates the symptoms of IVL and increases the risk of operation. High venous flow caused by vascular malformation increases the right ventricular pressure (Cases 2 and 3). Timely embolization may significantly improve the problem of right ventricular overload, reverse right heart failure, promote the recovery of right ventricular systolic pressure, and improve the blood supply of the lungs (Table 4). The rich blood supply of tumors caused by vascular malformations may promote IVL tumor growth in addition to increasing the risk of surgical bleeding. As in Case 4, the IVL significantly blocks IVC and reaches the heart.

**Table 4:** Echocardiography of Case 2 and 3 before and after coil embolization.

**Variable**	**Reference range**	**Case 2**		**Case 3**	
		**Before**	**After**	**Before**	**After**
Right atrial vertical diameter	≤51 mm	83	80	87	79
Right atrial transveral diameter	≤41 mm	78	77	72	66
Right ventricle diameter	≤39 mm	63	53	NR	49
IVC diameter	≤21 mm	39	34	37	28
TAPSE	≥17 mm	11	18	21	16
Tricuspid regurgitation velocity		2.5	2.8	2.5	2.8

According to statistics, the recurrence rate of intravascular tumor residue was much greater than that of patients with clean intravascular excision (Table 3). This discrepancy could be attributable to the bias introduced by the short sample size. Nevertheless, the ligation of the bilateral internal iliac vein and ovarian vein is helpful to prevent the recurrence or shedding of residual tumor embolus (26). Patients who have a pelvic tumor resection are more likely to experience complications than patients who do not have a pelvic tumor resection (Table 3), which could be owing to the abundance of the pelvic vascular bed, or possibly the creation of tumor-related arteriovenous malformations.

There is currently no consensus on the diagnosis and treatment of IVL, and only a few retrospective cohort studies in big centers with more cases were conducted (10, 27). Ma et al. presented an IVL staging system, categorizing IVLs into stages 1 through 4 based on the extent of intravenous tumor spread (28). Liu et al. categorized IVLs that enter the heart chambers into types 1–5 and discussed surgical plans for each type of IVLs (29). Further classification methods and surgical techniques were discussed by Li et al., although the surgical categorization differs from that suggested by Liu et al. According to the relative diameter between tumor and IVC, Li et al. recommended four types of surgery. Such classification agrees with Ma et al.'s method on stage 3 IVLs, including IVLs reaching and not reaching the right atrium. However, effective imaging tools are still needed to determine whether the tumor in the heart can be retrieved from the IVC in advance according to their reports. Therefore, different institutes should share their surgical experiences and promote diagnostic guidelines to decrease misdiagnosis and missed diagnoses, improve complication management, and decrease significant complications and recurrence.

### Limitations and Conclusions

To be clear, our study on IVL complicated by AVF, case series, and literature review has several limitations. First, the cases in the study were mainly from one medical center, some patients may have been overlooked in other places due to the rarity of this condition. The study was retrospective, providing less powerful evidence than prospective studies.

The symptoms of patients with IVL, caused by mass blocking venous reflux, mainly resemble that of right ventricular dysfunction and are easily misdiagnosed due to lack of experience or insufficient imaging examination. Incomplete intravascular IVL resection might contribute to recurrence; therefore, physicians should try to remove the tumor from the vessels completely. Some patients who had IVL complicated by pelvic AVF experienced a higher risk of bleeding in surgery, indicating the importance of dealing with the AVF in advance. Further studies on the above problems are necessary.

We point out that IVL combined with AVF aggravates congestion symptoms of peripheral organs, such as leg edema, ascites, hepatomegaly, jaundice, and intestinal bleeding, which can be seen in our cases and in the literature. Embolization of AVF in advance may reduce the risk of bleeding in IVL surgery. For patients with symptoms of right heart failure after pelvic surgery, imaging with a contrast agent (MRA and DSA) should be recommended to eliminate potential AVFs. In our cases, coil embolization was performed, the right ventricular function improved, and the short-term effect was satisfactory. Moreover, considering the possibility of iatrogenic AVF in patients with IVL, the tumor should be removed carefully, with attention to fine anatomical structure when ligating blood vessels, avoid ligation of arteries and veins together, and reduce secondary arteriovenous malformations.

## Data Availability Statement

The original contributions presented in the study are included in the article/Supplementary Material, further inquiries can be directed to the corresponding authors.

## Ethics Statement

The study protocol was approved by the Research and Ethics Board of the Peking Union Medical College Hospital. The patients/participants provided their written informed consent to participate in this study and for the publication of any potentially identifiable data included in this article.

## Author Contributions

HK and YCa analyzed the patient data and were major contributors in writing the manuscript. YZ and YCh made substantial contributions to the study design and gave final approval to the version to be published. All authors read and approved the final manuscript.

## Funding

This research was funded by CAMS Innovation Fund for Medical Sciences (CIFMS, numbers 2021-I2M-C&T-A-006 and 2021-I2M-1-016), the Major Research Program of Natural Science Foundation of China (51890892), the National Natural Science Foundation of China (NSFC, numbers 82070492, 81770481, and 82170516), and the Non-profit Central Research Institute Fund of Chinese Academy of Medical Sciences (2021-JKCS-027).

## Conflict of Interest

The authors declare that the research was conducted in the absence of any commercial or financial relationships that could be construed as a potential conflict of interest.

## Publisher's Note

All claims expressed in this article are solely those of the authors and do not necessarily represent those of their affiliated organizations, or those of the publisher, the editors and the reviewers. Any product that may be evaluated in this article, or claim that may be made by its manufacturer, is not guaranteed or endorsed by the publisher.
