# Supplementary material for: Intravenous Leiomyomatosis Complicated by Arteriovenous Fistula: Case Series and Literature Review
Source: Front Cardiovasc Med. 2022 Jun 13;9:878386. doi: 10.3389/fcvm.2022.878386 (PMC9234661; doi:10.3389/fcvm.2022.878386)
Supplement: Supplementary file 1 [file Data_Sheet_1.docx]

| **Supplemental Table 1. Characteristics of the included studies** | | | |
| --- | --- | --- | --- |
| Study | Region | Number of cases | Reference |
| Van Maercke et al. 2020 | Belgium | 1 | [1] |
| Zhang et al. 2012 | China | 1 | [2] |
| Cornélis et al. 2012 | France | 1 | [3] |
| Schindle et al. 2012 | USA | 2 | [4] |
| Lou et al. 2011 | China | 1 | [5] |
| Lee et al. 2011 | Korea | 1 | [6] |
| Barksdale et al. 2011 | USA | 1 | [7] |
| Yu et al. 2011 | China | 2 | [8] |
| Fang et al. 2010 | China | 1 | [9] |
| Anselmi et al. 2010 | Italy | 6 | [10] |
| Galajda et al. 2010 | Hungary | 1 | [11] |
| Worley et al. 2009 | USA | 4 | [12] |
| Deac et al. 2009 | UK | 1 | [13] |
| Singh et al. 2010 | Australia | 1 | [14] |
| Vaquero et al. 2009 | USA | 2 | [15] |
| Bodner-Adler et al. 2009 | Australia | 1 | [16] |
| Liu et al. 2009 | China | 1 | [17] |
| Wu et al. 2009 | China | 1 | [18] |
| Leitman et al. 2008 | Israel | 2 | [19] |
| Lo et al. 2008 | China | 1 | [20] |
| Esmaeilzadeh et al. 2007 | Iran | 1 | [21] |
| Coganow et al. 2006 | USA | 1 | [22] |
| Wong et al. 2006 | China | 1 | [23] |
| Moorjani et al. 2005 | UK | 1 | [24] |
| Ozer et al. 2005 | Turkey | 1 | [25] |
| Elkington et al. 2005 | Australia | 1 | [26] |
| Topcuoglu et al. 2004 | Turkey | 1 | [27] |
| Ahmed et al. 2004 | Germany | 1 | [28] |
| Kocaoglu et al. 2003 | Turkey | 1 | [29] |
| Khayata et al. 2003 | Arab | 1 | [30] |
| Lo et al. 2001 | China | 1 | [31] |
| Wakiyama et al. 2000 | Japan | 1 | [32] |
| Kim et al. 2019 | Korea | 1 | [33] |
| Chiang et al. 2018 | China | 1 | [34] |
| Jalaguier-Coudray et al. 2017 | France | 3 | [35] |
| Price et al. 2017 | USA | 1 | [36] |
| Schäfer et al. 2017 | Switzerland | 1 | [37] |
| Thompson et al. 2016 | UK | 1 | [38] |
| Fogarty et al. 2017 | Australia | 1 | [39] |
| Caldentey et al. 2016 | Spain | 1 | [40] |
| Zhang et al. 2016 | China | 2 | [41] |
| Correia et al. 2016 | Portugal | 1 | [42] |
| Knight et al. 2017 | USA | 1 | [43] |
| Wang et al. 2016 | China | 1 | [44] |
| Jain et al. 2015 | India | 1 | [45] |
| Zhang et al. 2015 | China | 1 | [46] |
| Harnoy et al. 2016 | France | 1 | [47] |
| Li et al. 2016 | China | 1 | [48] |
| Efthimiadis et al. 2015 | Greece | 1 | [49] |
| Ribeiro et al. 2013 | Portugal | 1 | [50] |
| Stoleriu et al. 2013 | Germany | 1 | [51] |
| Oliveira et al. 2013 | Portugal | 1 | [52] |
| Baboci et al. 2014 | Albania | 1 | [53] |
| Matos et al. 2013 | Portugal | 1 | [54] |
| Zhang et al. 2013 | China | 2 | [55] |
| Hinojosa et al. 2013 | USA | 1 | [56] |
| Li et al. 2014 | China | 7 | [57] |
| Yu et al. 2018 | China | 8 | [58] |
| Carr et al. 2015 | USA | 14 | [59] |
| Arif et al. 2006 | UK | 1 | [60] |
| Corbett et al. 2020 | Ireland | 1 | [61] |
| Su et al. 2020 | China | 14 | [62] |
| Yano et al. 2020 | Japan | 1 | [63] |
| Xu et al. 2020 | China | 1 | [64] |
| Marrone et al. 2019 | Italy | 1 | [65] |
| Luo et al. 2019 | China | 5 | [66] |
| Luo et al. 2019 | China | 8 | [67] |
| Barnaś et al. 2019 | Poland | 1 | [68] |
| He et al. 2019 | China | 3 | [69] |
| Kommoss et al. 2019 | Germany | 1 | [70] |
| Wu et al. 2019 | China | 1 | [71] |
| Gao et al. 2020 | China | 1 | [72] |
| Qin et al. 2018 | China | 1 | [73] |
| Zhao et al. 2020 | China | 22 | [74] |
| Zhang et al. 2010 | China | 5 | [75] |
| Liu et al. 2018 | China | 50 | [76] |
| Yang et al. 2018 | China | 16 | [77] |
| Li et al. 2018 | China | 24 | [78] |

**Supplemental Table 1 Reference**

[1] Van Maercke J, Van Rompuy AS, Poppe W, Verbelen T, Delcroix M, Belge C. Intravascular Leiomyomatosis as a Rare Cause of Nonthrombotic Pulmonary Embolism. Case Rep Vasc Med. 2020 Jul 14;2020:6084061.

[2] Zhang C, Liu X, Ma G, et al. Pulmonary embolization as the primary clinical manifestation of intravenous leiomyomatosis with intracardiac extension. Ann Thorac Surg. 2012 Sep;94(3):1012.

[3] Cornélis F, Belleannée G, Lederlin M. Cardiac extension of an intravascular leiomyomatosis 43 years after hysterectomy. J Thorac Cardiovasc Surg. 2012 Jul;144(1):e3-5.

[4] Schindler N, Babrowski T, DeSai T, Alexander JC. Resection of intracaval leiomyomatosis using abdominal approach and venovenous bypass. Ann Vasc Surg. 2012 Jan;26(1):109.e7-11.

[5] Lou YF, Shi XP, Song ZZ. Intravenous leiomyomatosis of the uterus with extension to the right heart. Cardiovasc Ultrasound. 2011 Sep 24;9:25.

[6] Lee S, Kim DK, Narm KS, Cho SH. Pulmonary artery embolization of intravenous leiomyomatosis extending into the right atrium. Korean J Thorac Cardiovasc Surg. 2011 Jun;44(3):243-6.

[7] Barksdale J, Abolhoda A, Saremi F. Intravenous leiomyomatosis presenting as acute Budd-Chiari syndrome. J Vasc Surg. 2011 Sep;54(3):860-3.

[8] Yu L, Shi E, Gu T, Xiu Z, Fang Q, Wang C. Intravenous leiomyomatosis with intracardiac extension: a report of two cases. J Card Surg. 2011 Jan;26(1):56-60.

[9] Fang F, Lam YY. An unusual cause of right heart failure in a patient with previous hysterectomy. J Ultrasound Med. 2010 Nov;29(11):1647-50.

[10] Anselmi A, Tsiopoulos V, Perri G, Palladino M, Ferrante A, Glieca F. Case series of resection of pelvic leiomyoma extending into the right heart: surgical safeguards and clinical follow-up. J Cardiovasc Med (Hagerstown). 2010 Aug;11(8):583-6.

[11] Galajda Z, Copotoiu C, Suciu H, Tint D, Glasz T, Deac R. The diagnosis, morphological particularities, and surgical technique in a case of intravascular leiomyoma extended to the right heart chambers. J Vasc Surg. 2010 Apr;51(4):1000-2.

[12] Worley MJ Jr, Aelion A, Caputo TA, et al. Intravenous leiomyomatosis with intracardiac extension: a single-institution experience. Am J Obstet Gynecol. 2009 Dec;201(6):574.e1-5.

[13] Deac MO, Sheppard MN, Moat N, Burke SJ, Christmas T, Mohiaddin RH. Images in cardiovascular medicine. From uterus to pulmonary embolus: an uncommon association. Circulation. 2009 Jul 21;120(3):e16-9.

[14] Singh T, Lamont PM, Otton GR, Thomson DS. Intravenous leiomyomatosis with intracardiac extension: first reported case in Australia. Heart Lung Circ. 2010 Jan;19(1):50-2.

[15] Vaquero ME, Magrina JF, Leslie KO. Uterine smooth-muscle tumors with unusual growth patterns. J Minim Invasive Gynecol. 2009 May-Jun;16(3):263-8.

[16] Bodner-Adler B, Bartl M, Wagner G. Intravenous leiomyomatosis of the uterus with pulmonary metastases or a case with benign metastasizing leiomyoma? Anticancer Res. 2009 Feb;29(2):495-6.

[17] Liu H, Pan L, Shen K, et al. Magnetic resonance imaging is useful for diagnosis and evaluation of recurrent intravenous leiomyomatosis before surgery. Fertil Steril. 2009 Sep;92(3):1150-1152.

[18] Wu CK, Luo JL, Yang CY, Huang YT, Wu XM, Cheng CL, Chiang FT, Tseng CD. Intravenous leiomyomatosis with intracardiac extension. Intern Med. 2009;48(12):997-1001.

[19] Leitman M, Kuperstein R, Medalion B, et al. A highly unusual right atrial mass presented in two women. Eur J Echocardiogr. 2008 Nov;9(6):833-4.

[20] Lo KW, Yu MY, Cheung TH. Low-grade endometrial stromal sarcoma with florid intravenous component. Gynecol Obstet Invest. 2008;66(1):8-11.

[21] Esmaeilzadeh M, Tavakolli A, Safaei A. Recurrent intracardiac leiomyomatosis. Can J Cardiol. 2007 Nov;23(13):1085-6.

[22] Coganow M, Das BM, Chen E, Crestanello JA. Single-stage resection of a mixed endometrial stromal sarcoma and smooth muscle tumor with intracardiac and pulmonary extension. Ann Thorac Surg. 2006 Oct;82(4):1517-9.

[23] Wong YY, Chu WC, Lam WW. Intravenous leiomyomatosis: computed tomography diagnosis. Hong Kong Med J. 2006 Jun;12(3):239-40.

[24] Moorjani N, Kuo J, Ashley S, Hughes G. Intravenous uterine leiomyosarcomatosis with intracardial extension. J Card Surg. 2005 Jul-Aug;20(4):382-5.

[25] Ozer N, Engin H, Akgül E, et al. An unusual case of recurrent mass in the right atrium: intravenous leiomyomatosis. Echocardiography. 2005 Jul;22(6):514-6.

[26] Elkington NM, Carlton M. Recurrent intravenous leiomyomatosis with extension up the inferior vena cava. Aust N Z J Obstet Gynaecol. 2005 Apr;45(2):167.

[27] Topcuoglu MS, Yaliniz H, Poyrazoglu H, et al. Intravenous leiomyomatosis extending into the right ventricle after subtotal hysterectomy. Ann Thorac Surg. 2004 Jul;78(1):330-2.

[28] Ahmed M, Zangos S, Bechstein WO, Vogl TJ. Intravenous leiomyomatosis. Eur Radiol. 2004 Jul;14(7):1316-7.

[29] Kocaoglu M, Bulakbasi N, Ugurel MS, Ors F, Tayfun C, Ucoz T. Value of magnetic resonance imaging in the depiction of intravenous leiomyomatosis extending to the heart. J Comput Assist Tomogr. 2003 Jul-Aug;27(4):630-3.

[30] Khayata GM, Thwaini S, Aswad SG. Intravenous leiomyomatosis extending to the heart. Int J Gynaecol Obstet. 2003 Jan;80(1):59-60.

[31] Lo KW, Lau TK. Intracardiac leiomyomatosis. Case report and literature review. Arch Gynecol Obstet. 2001 Jan;264(4):209-10.

[32] Wakiyama H, Sugimoto T, Ataka K, Yamashita C, Tsuji Y, Nakagiri K, Inoue K, Okada M. Intravenous leiomyomatosis extending into the right ventricular cavity: one-stage radical operation using cardiopulmonary bypass--a case report. Angiology. 2000 Jun;51(6):505-9.

[33] Kim JH, Baek JH. A Challenging Case of Intracardiac Leiomyomatosis Accompanied by Pseudo-Meigs Syndrome Originating from Uterine Leiomyoma. Ann Vasc Surg. 2019 Feb;55:309.e5-309.e8.

[34] Chiang CS, Chen PL, Kuo TT, Chen IM, Wu NY, Chang HH. One-stage surgery for removal of intravascular leiomyomatosis extending to right ventricle. Medicine (Baltimore). 2018 Mar;97(11):e0051.

[35] Jalaguier-Coudray A, Allain-Nicolai A, Thomassin-Piana J, Villard-Mahjoub R, Delarbre B, Rua S, Lambaudie E, Houvenaeghel G. Radio-surgical and pathologic correlations of pelvic intravenous leiomyomatosis. Abdom Radiol (NY). 2017 Dec;42(12):2927-2932.

[36] Price JD, Anagnostopoulos C, Benvenisty A, Kothuru RK, Balaram SK. Intracardiac Extension of Intravenous Leiomyomatosis. Ann Thorac Surg. 2017 Feb;103(2):e145-e147.

[37] Schäfer HM, Isaak A, Gürke L. Case report of an intracaval leiomyomatosis 10 months after complete hysterectomy. Int J Surg Case Rep. 2017;35:1-3. doi: 10.1016/j.ijscr.2017.03.031. Epub 2017 Mar 31.

[38] Thompson AT, Desai A, Ford SJ, Gourevitch D. Uterine leiomyomatosis with intracardiac extension. BMJ Case Rep. 2016 Dec 20;2016:bcr2016218234.

[39] Fogarty SJ, Hart G, Nicklin J, Venkatesh B, Boyne N, Wong DC, Stone JL, Rainbird AJ. Intracardiac Leiomyomatosis - an Unusual Cause of Syncope in a Middle-Aged Woman. Heart Lung Circ. 2017 Apr;26(4):e22-e25.

[40] Caldentey G, Flores E, San Antonio R, Caixal G, Sánchez P. A rare cause of intracardiac mass. Int J Cardiol. 2016 Nov 15;223:91-92.

[41] Zhang G, Yu X, Lang J. Intravenous leiomyomatosis with inferior vena cava or intracardiac extension and concurrent bilateral multiple pulmonary nodules: A report of 2 cases. Medicine (Baltimore). 2016 Aug;95(35):e4722.

[42] Correia P, Castro A, Rocha A, Freitas D, Carnide C, Moutinho O. Pelvic Intravenous Leiomyomatosis - Case Report. Rev Bras Ginecol Obstet. 2016 Aug;38(8):412-5.

[43] Knight J, Phillips DP, Esper SA, Zeh HJ, Badhwar V, Subramaniam K. Paradoxical Tumor Embolism and Recurrent Intracardiac Mass From Uterine Intravenous Leiomyomatosis. J Cardiothorac Vasc Anesth. 2017 Apr;31(2):642-645.

[44] Wang HC, Wang YB, Chen XH, Cui LL. Uterine Intravenous Leiomyomatosis with Intracardiac Extension and Pulmonary Benign Metastases on FDG PET/CT: A Case Report. Korean J Radiol. 2016 Mar-Apr;17(2):289-94.

[45] Jain N, Rissam HK, Mittal UK, Sharma A. Intravenous leiomyomatosis with intracardiac extension: an unusual presentation of uterine leiomyoma and evaluation with 256-slice dual-source multidetector CT and cardiac MRI. BMJ Case Rep. 2015 Dec 7;2015:bcr2015211712.

[46] Zhang AQ, Xue M, Wang DJ, Nie WP, Xu DB, Guan XM. Two-stage resection of a disseminated mixed endometrial stromal sarcoma and smooth muscle tumor with intravascular and intracardiac extension. Taiwan J Obstet Gynecol. 2015 Dec;54(6):776-9.

[47] Harnoy Y, Rayar M, Levi Sandri GB, Zamreek A, Turner K, Sulpice L, Boudjema K, Meunier B. Intravascular Leiomyomatosis with Intracardiac Extension. Ann Vasc Surg. 2016 Jan;30:306.e13-5.

[48] Li YQ, Yin XP, Xu ZW. Orthostatic hypotension and right heart failure as the initial manifestation of intravenous leiomyomatosis. Cardiol Young. 2016 Mar;26(3):586-8.

[49] Efthimiadis C, Petousis S, Grigoriou M, Ioannidis A, Tzouveleki I, Margioula-Siarkou C, Kalogiannidis I. Successful multiple-step management of intravenous leiomyomatosis diagnosed after episode of acute abdominal pain: Case report and review of literature. Int J Surg Case Rep. 2015;14:176-8.

[50] Ribeiro V, Almeida J, Madureira AJ, et al. Intracardiac leiomyomatosis complicated by pulmonary embolism: a multimodality imaging case of a rare entity. Can J Cardiol. 2013 Dec;29(12):1743.e1-3.

[51] Stoleriu C, Rizas K, Gawaz M, Geisler T. Intracaval and intracardiac leiomyomatosis of uterine origin. BMJ Case Rep. 2013 Nov 5;2013:bcr2012008368.

[52] Oliveira L, Ramos S. Anesthetic approach for a clinical case of intravenous leiomyomatosis: Case report. Braz J Anesthesiol. 2013 Nov-Dec;63(6):504-7.

[53] Baboci A, Prifti E, Xhabija N, Alimehmeti M. Surgical removal of an intravenous leiomyoma with intracardiac extension and pulmonary benign metastases. Heart Lung Circ. 2014 Feb;23(2):174-6.

[54] Matos AP, Ramalho M, Palas J, Herédia V. Heart extension of an intravenous leiomyomatosis. Clin Imaging. 2013 Mar-Apr;37(2):369-73.

[55] Zhang Y, Zhu J, Wang C, Tu R, Jiang J, Lu W. Multimodality treatment of two cases of intracardiac leiomyomatosis with enormous mass in the abdominopelvic cavity. Expert Rev Anticancer Ther. 2013 Feb;13(2):137-41.

[56] Hinojosa CA, Medina-Franco H, Orozco-Zepeda V, Martinez Mijangos O, Valdes KA, Aragon Han P. Infrarenal transcaval extraction of intracardiac leiomyomatosis. Ann Vasc Surg. 2013 Feb;27(2):238.e1-4.

[57] Li R, Shen Y, Sun Y, Zhang C, Yang Y, Yang J, Su R, Jiang B. Intravenous leiomyomatosis with intracardiac extension: echocardiographic study and literature review. Tex Heart Inst J. 2014 Oct 1;41(5):502-6.

[58] Yu HY, Tsai HE, Chi NH, Kuo KT, Wang SS, Chen CA, Chen YS. Long-term outcomes of surgical treatment for intravascular leiomyomatosis. J Formos Med Assoc. 2018 Nov;117(11):964-972.

[59] Carr RJ, Hui P, Buza N. Intravenous leiomyomatosis revisited: an experience of 14 cases at a single medical center. Int J Gynecol Pathol. 2015 Mar;34(2):169-76.

[60] Arif S, Ganesan R, Spooner D. Intravascular leiomyomatosis and benign metastasizing leiomyoma: an unusual case. Int J Gynecol Cancer. 2006 May-Jun;16(3):1448-50.

[61] Corbett GA, O'Gorman C, Kamran W. Intravenous leiomyomatosis: the first reported case of intraoperative intracaval embolisation of tumour to the right atrium. BMJ Case Rep. 2020 Mar 12;13(3):e233341.

[62] Su Q, Zhang X, Zhang H, et al. Intravenous Leiomyomatosis of the Uterus: A Retrospective Single-Center Study in 14 Cases. Biomed Res Int. 2020 Feb 14;2020:9758302.

[63] Yano M, Katoh T, Nakajima Y, et al. Uterine intravenous leiomyomatosis with an isolated large metastasis to the right atrium: a case report. Diagn Pathol. 2020 Jan 11;15(1):4.

[64] Xu Y, Gao X, Yang C, Liu J, Jin B, Shang D. Intravascular Leiomyomatosis Extending to Right Atrium: A Rare Caused Syncope. Ann Vasc Surg. 2020 May;65:287.e7-287.e10.

[65] Marrone G, Crinò F, Morsolini M, Caruso S, Miraglia R. Multidisciplinary approach in the management of uterine intravenous leiomyomatosis with intracardiac extension: case report and review of literature. J Radiol Case Rep. 2019 Jul 31;13(7):1-13.

[66] Luo G, Pan H, Bi J, et al. Surgical treatment of intravenous leiomyomatosis involving the right heart: a case series. J Int Med Res. 2019 Jul;47(7):3465-3474.

[67] Luo X, Li R, Li Z. Combined transthoracic echocardiography and contrast-enhanced ultrasonography to trace intravenous leiomyomatosis with intracardiac extension. Echocardiography. 2019 Aug;36(8):1573-1576.

[68] Barnaś E, Raś R, Skręt-Magierło J, et al. Natural history of leiomyomas beyond the uterus. Medicine (Baltimore). 2019 Jun;98(25):e15877.

[69] He J, Chen ZB, Wang SM, et al. Intravenous leiomyomatosis with different surgical approaches: Three case reports. World J Clin Cases. 2019 Feb 6;7(3):347-356.

[70] Kommoss F, Ebel T, Drusenheimer J, et al. Die intravenöse Leiomyomatose [Intravenous leiomyomatosis]. Pathologe. 2019 Feb;40(1):80-84. German.

[71] Wu YH, Lee YT, Lee CI, Tzeng YH, Wei J. Nonthrombotic pulmonary embolism caused by intravenous leiomyomatosis: A case report. Medicine (Baltimore). 2019 Jan;98(3):e14118.

[72] Gao B, Zhou D, Qian X, Zhang W, Ying L, Wang W. Primary leiomyoma of the inferior vena cava mimicking a cystic neoplasm of the pancreas: a case report. Cardiovasc Pathol. 2020 May-Jun;46:107097.

[73] Qin X, Liang W, Yue H, et al. Intravenous leiomyomatosis with extension to the pulmonary artery associated with syncope. J Card Surg. 2018 Nov;33(11):753-755.

[74] Zhao Y, Huang ZH, Fu W, Liu TS, Dong R. [Clinical analysis of intravenous-cardiac leiomyomatosis]. Zhonghua Yi Xue Za Zhi. 2020 Jun 9;100(22):1741-1744. Chinese.

[75] Zhang C, Miao Q, Liu X, et al. Intravenous leiomyomatosis with intracardiac extension. Ann Thorac Surg. 2010 May;89(5):1641-3.

[76] Liu J, Liang M, Ma G, et al. Surgical treatment for intravenous-cardiac leiomyomatosis. Eur J Cardiothorac Surg. 2018 Sep 1;54(3):483-490.

[77] Yang C, Fang H, Yang Y, et al. Diagnosis and surgical management of inferior vena cava leiomyomatosis. J Vasc Surg Venous Lymphat Disord. 2018 Sep;6(5):636-645.

[78] Li H, Xu J, Lin Q, et al. Surgical treatment strategies for extra-pelvic intravenous leiomyomatosis. Orphanet journal of rare diseases. 2020;15(1):153.
